# Human-Guided Learning for Probabilistic Logic Models

**DOI:** 10.3389/frobt.2018.00056

**Published:** 2018-06-25

**Authors:** Phillip Odom, Sriraam Natarajan

**Affiliations:** ^1^Georgia Tech Research Institute, Georgia Institute of Technology, Atlanta, GA, United States; ^2^University of Texas at Dallas, Dallas, TX, United States

**Keywords:** statistical relational learning, advice-giving, knowledge-based learning, structure learning, privileged information

## Abstract

Advice-giving has been long explored in the artificial intelligence community to build robust learning algorithms when the data is noisy, incorrect or even insufficient. While logic based systems were effectively used in building expert systems, the role of the human has been restricted to being a “mere labeler” in recent times. We hypothesize and demonstrate that probabilistic logic can provide an effective and natural way for the expert to specify domain advice. Specifically, we consider different types of advice-giving in relational domains where noise could arise due to systematic errors or class-imbalance inherent in the domains. The advice is provided as logical statements or privileged features that are thenexplicitly considered by an iterative learning algorithm at every update. Our empirical evidence shows that human advice can effectively accelerate learning in noisy, structured domains where so far humans have been merely used as labelers or as designers of the (initial or final) structure of the model.

##  1. Introduction

Machine learning and data mining have made significant progress in recent years, but most supervised learning techniques learn only from labeled data (Mitchell, 1997; Cristianini and Shawe-Taylor, 2000; Schapire and Freund, 2012). Hence, in spite of the successes in several real tasks, most of these methods consider the human (domain) expert to be a mere “labeler”. They fail to utilize the rich (possibly general) domain knowledge that experts are capable of providing. A key reason is that these methods employ an underlying propositional representation, i.e., they learn from a set of flat feature vectors. However, much of domain knowledge is typically more general. For example, when providing advice about driving, it is natural to say something like “do not change lanes if there is no vehicle in front of you”. Note that this advice can be easily represented in first-order logic using quantifiers. Similar advice can be provided in a variety of inherently relational domains such as Electronic Health Records, streaming data, organizational or social networks, and many more.

It must be mentioned that incorporating prior knowledge in machine learning has been explored by a small community. Most closely related to our work are knowledge-based neural networks (Towell and Shavlik, 1994), knowledge-based support vector machines (Fung et al., 2002) and their recent adaptations (Kunapuli et al., 2010; Kunapuli et al., 2013) that have explored the combination of knowledge and data to handle systematic noise. While specific adaptations differ, all these methods take as advice propositional horn clauses and convert them to their corresponding representation. These systems thus restrict the advice to a specific set of features instead of quantifying over all/some objects in the domain. In contrast, we aim to better exploit the advice (as horn clauses) in its natural formulation, i.e., as first-order logic statements.

To this effect, we employ the recently successful (probabilistic) methods that can directly operate on relational data called Probabilistic Logic Models (Getoor and Taskar, 2007) (PLMs). The advantage of PLMs is that they can succinctly represent probabilistic dependencies among the attributes of different related objects, leading to a compact representation of learned models. While effective, these algorithms for PLMs are mainly either data or knowledge-intensive. Data-intensive learning for PLMs learns purely from the data and effectively reduces the domain expert to simply providing training examples. Alternatively, knowledge-intensive learning can be inflexible as only the quantitative parameters are learned while the entire qualitative structure is generally defined by the expert. In this work, we aim to learn PLMs in both a data-intensive and a knowledge-intensive manner.

As mentioned earlier, domain experts are capable of providing significantly more expressive advice that can not be expressed in propositional frameworks. One of the key attractive features of PLMs is that the relationships between objects are specified using first-order logic. This could potentially allow for domain experts to express generalized knowledge (advice) about the domain. Examples include “People with family members that have a high risk of a disease d are also more likely to have a high risk of d” and “Movies with comedians are more likely to be of the genre comedy”.

Specifically, we consider a recently successful PLM learning algorithm called Relational Functional Gradient Boosting (RFGB) (Natarajan et al., 2012) as the underlying base learner and provide a framework^1^ for incorporating human advice. Our advice consists of preferred (labels that should have higher probability than other labels) and avoided target labels defined over spaces of examples. The space of examples where the advice applies is given by horn clauses which can be thought of as if-then rules specified by experts. Since our underlying representation is also based on first-order logic, the system can more faithfully exploit the advice compared to converting it to a different representation internally by the learning algorithm.

We show the versatility of our proposed framework along two directions. Firstly, we show that the proposed framework is not restricted to a particular type of PLM model. Specifically, we show that several formalisms such as Markov Logic Networks (MLNs) (Domingos and Lowd, 2009) and Relational Dependency Networks (RDNs) (Neville and Jensen, 2007) and tasks such as relational imitation learning (Natarajan et al., 2011) and relational transfer learning can benefit from this framework. Our framework uses both the advice and the data throughout the learning process and allows for sequential interaction where the expert could potentially add more advice as learning progresses.

Secondly, we demonstrate (both theoretically and empirically) that our formulation is applicable across different types of advice.

1. *Preferential advice*, inspired from preference elicitation approaches (Boutilier, 2002) that can improve learning in domains with systematic noise (where certain types of examples are consistently mislabeled possibly due to human or sensor error).
2. *Cost-based advice* that has been demonstrated to improve learning in domains with class-imbalance (essentially all relational domains since most relations such as friends of, advised by, married to are false).
3. *Qualitative constraints* such as monotonicity that have previously been shown to accelerate learning of probabilistic models in data-scarce and knowledge-rich domains (Altendorf et al., 2005; Yang and Natarajan, 2013).
4. *Privileged information*, a formulation developed by Vapnik and Vashist (2009) for SVMs where there are some features that are observed during training but not during testing and hence these features should be used to guide the learning algorithm to a better model.

**Figure 1:**
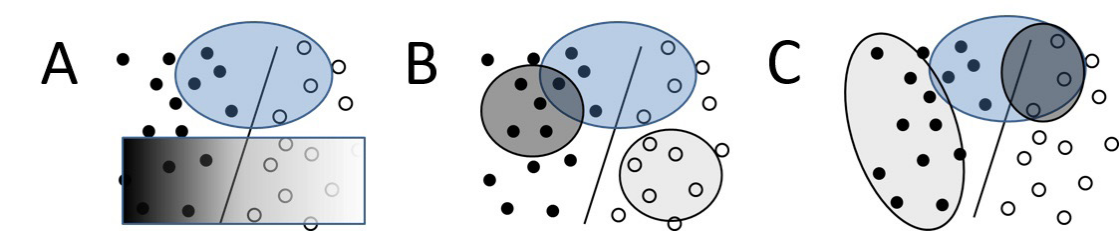
Three types of advice. Filled-in circles show positive examples while open circles show negative examples. General advice **(A)** is given over an arbitrary space of examples, false positive/false negative trade-off **(B)** is given over false positives and false negatives, and monotonicities **(C)** vary in strength across a single variable.

Several types of advice, capturing different information, are graphically depicted in Figure 1. The key idea here is to note that each type of advice can be understood as defining a constraint over the space of examples (in a manner similar to the original knowledge-based neural networks [Towell and Shavlik, 1994) and knowledge-based support vector machines (Fung et al., 2002)]. This interpretation allows us to develop a unifying framework based on preferences that can be effectively combined with the gradient-boosting learning technique.

As mentioned earlier, we evaluate the approach on different models and tasks. As far as we are aware, ours is the first work to explore the use of these advice types in the context of learning PLMs.

There has been a few successful adaptations of our proposed approach, specifically in the context of relation extraction and some healthcare problems. Odom et al. (2015a) adapted the preferences-based advice to extract the mentions of adverse drug events from medical abstracts. Soni et al. (2016) extended this work to a more general relation extraction task from text documents. MacLeod et al. (2016) adapted the class imbalance approach for learning to predict rare diseases from survey data. A similar approach was taken by Natarajan et al. (2017) to predict postpartum depression from survey data.

In summary, we make the following contributions - (1) We present the first work on exploiting human advice while learning several PLMs; (2) We outline a natural framework for the human to provide general advice about the domain as well as specific advice about particular examples; (3) We demonstrate the generality of our approach by presenting four types of advice that our framework is able to capture; (4) We show that our method naturally integrates human input with examples in a boosting formalism; and (5) Finally, through our experiments on a several data sets, we demonstrate that the framework efficiently exploits the human advice in learning effective models.

The rest of the paper is organized as follows: Section 2 introduces the background of PLMs/advice and discusses related work, Section 3 presents our advice-based framework for PLMs, Section 4 validates our framework empirically, and finally, Section 5 concludes and discusses future research directions.

##  2. Background/Related Work

###  2.1. Structure Learning of PLMs

Early approaches (Getoor et al., 2001a; Getoor et al., 2001b; Kersting and Raedt, 2007) to learning PLMs used two stages (Raedt and Kersting, 2004): the first step involved learning ‘pure logic’ (non-probabilistic) rules, which were unweighted as they had no probabilities associated with them. The second step involved learning the parameters (i.e., the weights or probabilities) of these rules. These two steps were initially performed separately, with the former typically based on rule-learning approaches such as Inductive Logic Programming (Lavrac and Dzeroski, 1993; Kersting and Raedt, 2007) or a graph search (Getoor et al., 2001b), and the latter based on graphical model techniques such as expectation maximization and gradient descent (Koller and Pfeffer, 1997; Kameya and Sato, 2000; Getoor et al., 2001b; Kersting and Raedt, 2007; Jaeger, 2008). Recently, there is an emphasis in adapting the “structure learning” methods from propositional graphical models for PLMs, specifically for the case of Markov Logic Networks (MLNs) (Mihalkova and Mooney, 2007; Biba et al., 2008; Kok and Domingos, 2009; Kok and Domingos, 2010). All these methods obtain the candidate clauses first, learn the parameters, score the weighted clauses given data and modify the clauses accordingly. This is a cumbersome process, as it requires repeated parameter learning which in turn requires repeated inference in its inner loop.

Consequently, there has been research on efficient structure learning (Natarajan et al., 2012) called Relational Function Gradient Boosting (RFGB) that seeks to learn the structure and parameters simultaneously which we present next.

###  2.2. Relational Functional-Gradient Boosting

Recall that the standard gradient descent learning algorithm starts with initial parameters $\theta_{0}$ and computes the gradient of the log-likelihood function [$\Delta_{1}=\frac{\partial }{\partial \theta }\,\log P\,\left(𝐗;\theta_{0}\right)$]. Friedman (2001) proposed an alternate approach to perform gradient descent where the log-likelihood function is represented using a regression function ψ over the examples 𝐱 and the gradients are performed with respect to this ψ⁢(x).

Functional gradient boosting starts with an initial function $\psi_{0}$ and iteratively adds gradients $\Delta_{m}$. Each gradient term ($\Delta_{m}$) is a regression function over the training examples and the gradients at the $m^{t\,h}$ iteration can be represented as $\left\langle x_{i},\Delta_{m}\,\left(x_{i}\right)\right\rangle$ where $x_{i}\in$ training examples. Also, rather than directly using $\left\langle x_{i},\Delta_{m}\,\left(x_{i}\right)\right\rangle$ as the gradient function, functional gradient boosting *generalizes* by fitting a regression function ${\hat{\psi }}_{m}$ (generally regression trees) to the gradients $\Delta_{m}$. The final model $\psi_{m}=\psi_{0}+{\hat{\psi }}_{1}+\cdots +{\hat{\psi }}_{m}$ is then a sum over these regression trees.

This method has been extended to various relational models for learning the structure (Kersting and Driessens, 2008; Karwath et al., 2008; Natarajan et al., 2011; Natarajan et al., 2012). The examples are ground atoms of the target predicate [for example, workedUnder(x, y)]. The ψ function is represented using relational regression trees (RRTs)(Blockeel, 1999). Since these are relational models, the ψ function depends on all the ground atoms and not just the grounding of the target predicate. For example, the probability function used by Natarajan et al. (2012) to learn the structure of Relational Dependency Networks (RDNs) (Neville and Jensen, 2007) was: $P\,\left(x_{i}\right)$= $s\,i\,g\,m\,o\,i\,d\,\left(\psi \,\left(x_{i};P\,a\,\left(x_{i}\right)\right)\right)$ where $P\,a\,\left(x_{i}\right)$ are all the relational/first-order logic facts that are used in the RRTs learned for $x_{i}$. They showed that the functional gradient of the likelihood for RDNs is

(1) $$\frac{\partial P(X=x)}{\partial \psi (x_{i})}=I(y_{i}=1)-P(y_{i}=1;x_{i},Pa(x_{i}))$$

which is the difference between the true distribution (I is the indicator function) and the current predicted distribution. For positive examples, the gradient is always positive and pushes the ψ function value ($\psi_{0}+\Delta_{1}+\cdots +\Delta_{m}$) closer to ∞ and the probability value closer to 1. Similarly the gradients for negative examples is negative and hence the ψ function is pushed closer to -∞ and probability closer to 0. While effective, this method is still data-intensive and treats humans as mere “labelers”. We use this method as our underlying learning method and show how to exploit human advice while learning.

####  2.3. Advice-Based Learning

Incorporating advice in propositional domains to improve the learning process has a rich history and has been explored in several directions (Towell and Shavlik, 1994; Kunapuli et al., 2010; Kunapuli et al., 2013). While specific adaptations differ, in all these methods, *a single piece of advice is defined over some set of the ground features or example space*. This space can then be labeled much like traditional examples. For example, Kunapuli et al. (2013) apply this idea to inverse reinforcement learning - where the goal is to learn a policy, a mapping from examples to actions. Intuitively their advice specifies a set of preferred actions that an agent should prefer selecting over a different set of avoided actions that the agent should avoid selecting for particular states.

Traditionally advice in PLMs has not been expressive and mainly consisted of hand-coding the structure and possibly even the parameters of the model. Such techniques have been used mainly in natural language processing tasks (Riedel et al., 2009; Yoshikawa et al., 2009; Poon and Vanderwende, 2010). While such techniques have been successful, the learning algorithm does not modify the structure of the model. As a result, they do not introduce potentially novel interactions based on the training data. A related method by Natarajan et al. (2013) used learned models from a source domain as initial models in the target domain and boosted the gradients based on examples from the target domain. However, if the training data is sub-optimal, it is possible that the learning algorithm will refine the model away from the advice (which is precisely the goal in transfer learning as the target domain is different from the source domain). On the other hand, we seek to use the advice throughout the learning process and thereby handle noisy examples.

Recently, the boosting algorithm was modified to handle *class-imbalance* using human advice (Yang et al., 2014). The key idea is to use human advice to specify the trade-off between false positives and negatives compared with the standard method of sub-sampling or over-sampling examples of a particular class (Chawla, 2005). This advice was then converted into constraints for the learning algorithm. This work is quite similar to our proposed approach. However, our advice is more general in terms of preferences and as we show both empirically and theoretically, our proposed formalism can subsume the class-imbalance trade-off case.

We go beyond this trade-off and demonstrate how more types of advice such as *qualitative constraints* (e.g., monotonicity or synergy) can be modeled in our formalism. While there has been work on learning with qualitative constraints for Bayesian networks (Altendorf et al., 2005; Yang and Natarajan, 2013), they have not yet been applied to the relational case. Our approach is the first-of-its-kind advice-taking method for general PLMs.

A different, but related type of advice that we consider is the use of *privileged information* (Vapnik and Vashist, 2009; Vapnik and Izmailov, 2015), where some advice is available during training time that is not present during evaluation/deployment or testing times. The idea is that this is similar to learning in a classroom where the teacher could provide subtle hints while teaching the classes but these hints may not be directly used during the exams. These hints however guide the student to learning a better model. While the original work employed these hints as constraints when learning a SVM, we extend this to the relational setting.

Our work can be seen as an unifying approach that entails different types of advice - preferences, class imbalance constraints, qualitative constraints and privileged information. We build upon the successful gradient-boosting technique to build this framework that treats human as more than a mere labeler.

##  3. Advice-Based Learning in Relational Problems

As mentioned earlier, learning in PLMs in spirit is similar to propositional graphical models and has two components - parameter learning and structure learning. Structure learning identifies the presence of a qualitative (influential) relationship between logical predicates while the parameters quantify the strength of these relationships. Most of the structure learning in literature uses an initial model from the expert and uses the data to fix the mistakes in these models. This essentially fixes the mistake in the advice and does not explicitly model the systematic errors in the training instances. This can lead to an inferior performance on noisy examples.

To address this issue, we now present a unifying framework for advice-based learning that is able to effectively exploit multiple types of advice to fix noisy training data in the form of *label preferences, qualitative constraints, class imbalance* and *privileged information*. One of the key contributions of this work is to demonstrate that label preferences can subsume many other types of advice. Our label preferences consist of a set of preferences over the labels specified by the user and motivated by the successful *preference elicitation* (Boutilier, 2002; Walsh, 2007) for propositional domains. In relational domains, as we show, obtaining these preferences is quite natural due to the generality of the underlying representation. Similar elicitation of other types of constraints such as qualitative constraints and class imbalance are natural and have been demonstrated considerably in propositional domains. We extend these to the relational setting. A related and different type of advice is that of privileged information (Vapnik and Vashist, 2009) that consists of a set of additional features specified (potentially by the user) over the training examples. These privileged features can potentially guide the learning process to better generalize over the standard features that will be incorporated into the model. We formalize a unifying framework that is able to integrate these different types of advice. To this effect, we adapt the function boosting approach with a modified objective function to utilize the human advice. We first introduce label preferences (which can easily model the qualitative constraints and class imbalance) and privileged information before discussing the learning algorithm. We use the term preferences to broadly consider label preferences, class imbalance and qualitative constraint advice pieces.

###  3.1. Advice via Preferences

We employ the use of first-order logic to capture the expert’s knowledge as a set of preference rules on the space of labels/actions as a function of the attributes of the object or related objects. Formally, our advice is defined as,

DEFINITION 1. *A relational advice set (RAS), *R* is specified as a set of relational advice rules (RAR), *$r_{1},r_{2},\ldots ,r_{A}$* and weights for labels that are preferred (*$\beta_{t}$*) and avoided (*$\beta_{f}$*) by the advice*^*2*^*. Each RAR, *$r_{a}$* is defined using the triple *⟨F,l+,l-⟩* where *F* is the relational advice constraint (RAC) clause specifying the subset of examples, *l+* is the weighted preferred label and *l-* is the weighted avoided label.*

DEFINITION 2. *A relational advice constraint (RAC), *F* is defined using a Horn clause *$\wedge_{i}f_{i}\,\left(x_{i}\right)\Rightarrow l\,a\,b\,e\,l\,\left(x_{e}\right)$*, where *$\wedge_{i}f_{i}\,\left(x_{i}\right)$* specifies the conjunction of conditions under which the advice applies on the example arguments *$x_{e}$*.*

**Figure 2:**
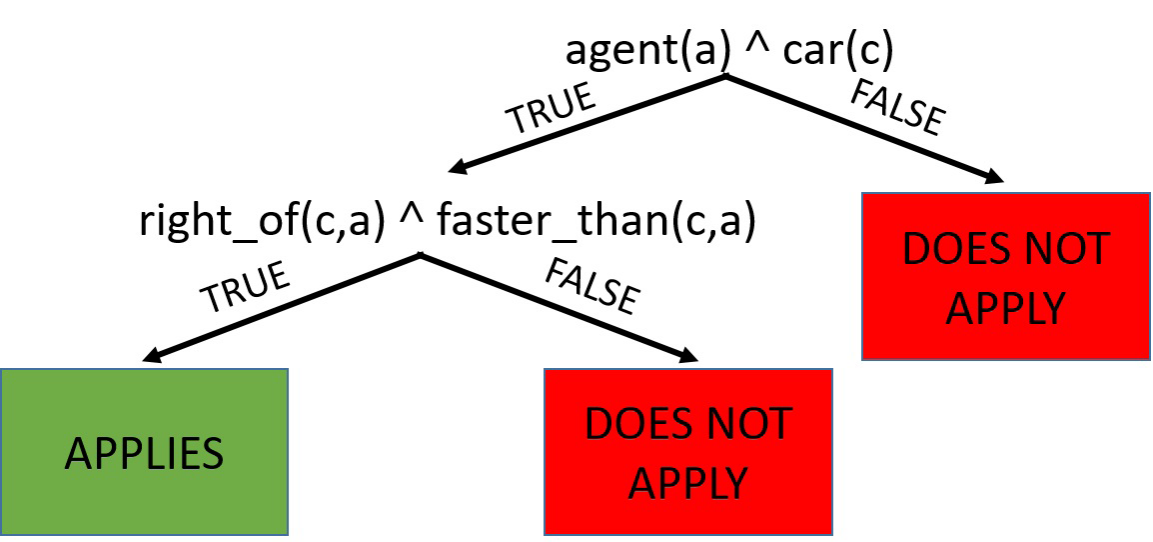
An advice model in the DRIVING domain for predicting lane changes on an interstate. Each node in the decision tree is a relational condition. The leaf nodes represent whether or not the advice will apply to examples that reach that leaf.

EXAMPLE 1. *Consider imitation learning in the driving domain, as used in **Figure 2**, where the task is to learn a policy to control a vehicle. This challenging task has a large potential state space and represents a stochastic problem where advice could be useful. A potential advice could be that if any car *c* passes an agent (car a) on the right, then it should move into the right lane. In this case, the advice set contains one rule, *$r_{1}=\langle F,l+,l-\rangle$* with *$\beta_{t}=$$\beta_{f}=1$*. Here *F=agent(a)∧car(c)∧right_of(c,a)∧faster_than(c,a),l+=move_right* and *l−=stay*.*

Given the label preferences, the goal is to learn a model that has higher probabilities for the preferred labels as compared to the avoided labels. Consider 𝐬 to be the set of training examples for which the advice is applicable, i.e., $𝐬=\left\{s_{i}|B,F⊢l\,a\,b\,e\,l\,\left(s_{i}\right)\right\}$, where B is the background knowledge and F is the advice constraint. The learned model should have a higher probability of the preferred target than the probability of the avoided target in the training examples, i.e., $\forall s_{i}\in 𝐬,P\,\left(l+\left(s_{i}\right)\right)\geq P\,\left(l-\left(s_{i}\right)\right)$. The magnitude of this constraint is determined by the weights on the preferred ($\beta_{t}$) and avoided ($\beta_{f}$) targets.

EXAMPLE 2. *For the example presented earlier, when any car *c* passes car *a* on the right, *P⁢(m⁢o⁢v⁢e⁢_⁢r⁢i⁢g⁢h⁢t⁢(a))≥P⁢(s⁢t⁢a⁢y⁢(a))*.*

Our advice can also handle sets of preferred and avoided labels by converting them into multiple advice rules for every pair of preferred and avoided labels. In our example, being passed on the right also suggests that the agent should not move into the left lane, i.e., l-={s⁢t⁢a⁢y,m⁢o⁢v⁢e⁢_⁢l⁢e⁢f⁢t}. We can also use RRTs (Blockeel, 1999) to specify the regions of the example space where advice is applicable. Since every path from root to leaf can be viewed as a Horn clause, a tree is a decision-list of Horn clauses. Figure 2 shows the relational advice constraints as a relational tree for our earlier example. The advice is applicable on examples which reach the green node and not applicable for examples reaching the red node.

We show the versatility of label preferences by showing how they can be used for cost-based learning as well to specify qualitative constraints.

**Class-Imbalance**: Class-imbalance is a common problem in relational learning as most relationships (e.g., friends or supervisors) are not true. These large imbalances can lead to predicting only the majority class. Recently, an algorithm was proposed (Yang et al., 2014) that showed how to incorporate advice about class-imbalance as a cost function inside the RFGB framework, quite similar to our approach here. While that particular approach can only handle the trade-off between false positives and false negatives, we show that our approach is more general and can handle their case too.

Advice for class-imbalance problems can be thought of as providing advice over the false positive and false negative regions. In order to be more sensitive to false positives and less sensitive to false negatives, we can simply provide advice to increase the probability of false positives and decrease the probability of false negatives. The $\beta_{t}$ parameter will give the weight for increasing the probability of false positives and the $\beta_{f}$ parameter will give the weight for decreasing the probability of false negatives. So by simply controlling the two βs in our framework, one can weigh the impact of false positives and false negatives differently.

As an example, consider the problem of predicting whether an actor will finish a movie or whether he will quit. It is important for production company to select an actor that will stick with the movie after it has invested significant resources. The corresponding advice would contain two rules. The first would “prefer” false positives while the other would “avoid” false negatives. As we show in our experiments, this approach yields similar results to published work on soft margin for RFGB (Yang et al., 2014). This clearly demonstrates that the preference-based advice can encompass the case of class-imbalance advice for PLMs.

**Qualitative Constraints**: Another rich type of advice, that we consider in this work, is the use of qualitative constraints (Kuipers, 1994) such as monotonicities and synergies. For instance, it is natural for a physician to explain that increased cholesterol levels elevate the risk of heart attack. This is a classic example of a monotonicity statement which essentially explains that P(hA=true|chol=high)>P(hA=true|chol=low) where h⁢A is the risk of heart attack. These statements can be interpreted as rules that provide the interaction between a set of attributes and their influence on a single target attribute. Such knowledge can be obtained easily for many real-tasks and has previously been employed in the context of learning probabilistic models (Altendorf et al., 2005; Yang and Natarajan, 2013) in propositional domains where data is scarce. We consider learning from such rich advice in relational domains.

Advice on qualitative constraints can be viewed as providing multiple pieces of advice over a given feature. As the value of that feature increase (or decreases), successively more advice will apply causing $n_{t}$ (or $n_{f}$) to scale based on that feature. Considering advice in this way gives a more accurate way of understanding natural relationships. For example, while “a movie with an actor that is a comedian is more likely to be of the genre comedy” is an accurate statement, it is very noisy as comedians may also appear in action or other movies. “The more comedians that act is a movie, the more likely that the movie is a comedy” is a much more accurate statement. It is exactly this kind of statement that monotonicities hope to capture. In this example, $n_{t}$ will scale with the number of actors in a movie that are considered comedians.

###  3.2. Advice via Privileged Information

While preferences are easier to understand and model, privileged information takes a different form of additional features that are available at training time, but not during testing. This additional information can be used to better generalize to unseen instances. Formally,

DEFINITION 3. *Relational privileged information (RP) is a set of features *$x^{\mathrm{RP}}=x^{\mathrm{PF}}\cup z$* where *z⊂x*. The features are specified in first-order logic over the training examples, but not available during testing.*

Unlike privileged information in propositional domains where privileged information (${𝐱}^{\mathrm{𝐏𝐅}}$) and the standard features (𝐱) are mutually exclusive, relational privileged information may include information from the standard features set (represented by 𝐳). This is due to the fact that some relational features do not provide much information themselves, but serve to “bridge” between other objects (e.g., friends or parent_of). When learning a clause, such relations by themselves will not increase the score of the search. Thus, these relations might never be picked when learning a relational model. Including them in the set of relational privileged information may be necessary to learn an effective model over the privileged features.

EXAMPLE 3. *Consider the same driving domain as in the previous section. Privileged information could include the local traffic patterns (e.g., rush hour, major/minor interchanges) or a description of the different nearby areas (e.g., demographics, business or residential).*

Privileged information can potentially guide or bias the learning algorithm to select features that generalize these concepts to other areas of the feature space. Privileged information provides another way for domain experts to interact with the learner. The RAC used to define the label’s preference can be viewed as a user-defined binary feature. We will empirically explore the connection between these different types of advice in the experimental section.

###  3.3. Knowledge-Based Objective Function

We aim to incorporate both preferences and privileged information into a unifying framework for knowledge-based relational learning. As mentioned earlier, one common way of introducing knowledge into relational learning is to use advice to define the initial structure or parameters (in this case the first tree of the boosted model) and refine this based on data. The key issue is that this method, in the worst case, can undo the advice to better fit the (possibly noisy) training instances. If the training examples are noisy, we do not want to fit only to the training examples but also utilize the advice. Hence, there is a necessity to faithfully and seamlessly integrate the advice into the learned model. We achieve this by modifying the objective function and including a cost that can account for each type of advice, allowing the learner to trade-off between the data and advice.

To achieve this, inspired by prior work of Gimpel and Smith (2010), we introduce a cost function c in the denominator of the log-likelihood function. While in their work, this was employed as a regularization term for a log-linear model, we employ this as a penalty term for violating the advice provided by the expert. This modified log-likelihood (MLL) function using the functional representation is given as,

(2) $$MLL(x,y)=\sum_{x_{i}\in x}\log\frac{\exp(\psi (x_{i};y_{i}))}{\sum_{y^{\prime }}\exp(\psi (x_{i};y^{\prime })+c(y_{i},y^{\prime },\psi ))}$$

Different types of advice require different cost functions and affect the learning process in different ways. While label preferences alter the predictions over a set of potentially noisy training examples, privileged information aims to improve generation by expressing similarities or differences among the feature space. We now explore the cost functions for both label preference $\left(c_{LP}\right)$ and privileged information ($c_{R\,P}$).

####  3.3.1. Label Preferences

In the case of label preferences, our cost function is used to penalize the model that does not fit to the advice. Since the cost function penalty depends only on the advice and the current model but not on the example labels y and $y^{\prime }$, we can redefine it as $c\,\left(x_{i},\psi \right)$. We define the cost function as

(3) $$c_{LP}(x_{i},\psi )=-\lambda \times \psi (x_{i})\times [\beta_{t}\times n_{t}(x_{i})-\beta_{f}\times n_{f}(x_{i})]$$

We use $n_{t}$ to indicate the number of advice rules that prefer the example to be true and $n_{f}$ to be the number of rules that prefer it to be false. The $\beta_{t}$ and $\beta_{f}$ weigh the magnitude that the advice should increase (or decrease) the probability of an example. By default both β parameters are set to 1, but these parameters are particularly useful when specifying advice to correct class-imbalance where errors in one class are more significant than errors in other classes^3^. We use λ to scale the cost function and $\psi \,\left(x_{i}\right)$ is the current value of the ψ function for the example $x_{i}$.

EXAMPLE 4. *Consider two advice rules:*

$r_{1}=\langle agent(a)\wedge car(b)\wedge right\_of(b,a)\wedge ,faster\_than(b,a)\;\;⟹\;label(a),move\_right,stay\rangle \;r_{2}=\langle agent(a)\wedge right\_of(c,a)\wedge traffic\_stop(c)\;\;⟹\;label(a),\{move\_left,stay\},move\_right\rangle$

**Table:** 

Examples	move_left		stay		move_right	
	$n_{t}$	$n_{f}$	$n_{t}$	$n_{f}$	$n_{t}$	$n_{f}$
$e_{f_{r_{1}},f_{r_{2}}}$	0	0	0	0	0	0
$e_{t_{r_{1}},f_{r_{2}}}$	0	0	0	1	1	0
$e_{f_{r_{1}},t_{r_{2}}}$	1	0	1	0	0	1
$e_{t_{r_{1}},t_{r_{2}}}$	1	0	1	1	1	1

The preceding table contains the values of $n_{t}$ and $n_{f}$ for various classes of examples. $e_{f_{r_{1}},f_{r_{2}}}$ represents the examples where both conditions for rules $r_{1}$ and $r_{2}$ are false (i.e., no car passing the agent on the right and no police traffic stop in the right lane), $n_{t}\,\left(e_{f_{r_{1}},f_{r_{2}}}\right)=n_{f}\,\left(e_{f_{r_{1}},f_{r_{2}}}\right)=0$ for all labels. $n_{t}$ values are shaded green and $n_{f}$ red. This example demonstrates several properties of our formalism: (1) advice only applies when it is relevant, (2) label preferences can contain sets of labels, and (3) the formalism can handle contradictory advice ($n_{t}$/$n_{f}$ are both non-zero).

Assuming that both $\beta_{t}$ and $\beta_{f}$ are 1, then intuitively when the example label is the preferred target in more advice rules than the avoided target, $n_{t}-n_{f}$ will be positive. Higher (positive) regression values will result in a lower (negative) cost function. Since a high positive regression value corresponds to higher probability of example being true, the cost function is lower when the regression function aligns with the advice. On the other hand, if the regression value is negative, the cost is positive since the regression function does not fully align with the advice. We will discuss varying $\beta_{t}$ and $\beta_{f}$ values in detail later in this section.

####  3.3.2. Relational Privileged Information

In the case of privileged information, we have two sets of examples. Thus, we learn a model over the standard features [P(y|x)] as well over the privileged features [$P_{D}\left(y|x^{\mathrm{𝐑𝐏}}\right)$]. The cost function depends on the KL-divergence between predictions made using the standard features and predictions made over the privileged features. The KL-divergence represents knowledge in the privileged space that is not available in the standard feature space. This additional information can allow the learning algorithm to select better features from which to generalize. We define the cost function as

(4) $$c_{RP}(x_{i},\psi )=-\lambda \times KL(P_{D}(y_{i}|x_{i}^{RP})||P(y_{i}|x_{i}))$$

Similar to the previous case, λ can be used to scale the relative effect of the privileged information. We now show an example where privileged information is potentially useful.

EXAMPLE 5. *Consider predicting whether a car will be pulled over in the driving domain. Two potential risk factors for being pulled over are speeding or driving a sports car.*

Intuitively, speeding is more likely to generalize well across different vehicle models. However, a small sample of drivers in New York City might suggest that sports cars are more likely to be pulled over. Privileged information that includes the local area (e.g., Manhattan) may allow the learning algorithm to reduce the chance of selecting sports cars due to bias in the training set.

We now use functional-gradient boosting to maximize our modified objective function (MLL).

$$MLL(x)=\sum_{i}\log\exp(\psi (x_{i};y_{i}))-\log(\sum_{y^{\prime }}\exp(\psi (x_{i};y^{\prime })-c(x_{i},\psi )))\;\;\;=\sum_{i}\log\exp\psi (x_{i};y_{i})-\log\sum_{y^{\prime }}\exp(c(x_{i},\psi ))\;\;\;=\sum_{i}\log P(y_{i},x_{i};\psi )+c(x_{i},\psi )$$

Note that c may be either $c_{L\,P}$, $c_{R\,P}$ or a combination of the two. We derive the function gradients for each cost function separately.

$$\frac{\partial MLL_{LP}(x)}{\partial \psi (x_{i};y_{i})}=\frac{\partial }{\partial \psi (x_{i};y_{i})}\log P(y_{i},x_{i};\psi )+\lambda \cdot \psi (x_{i})\cdot [\beta_{t}\cdot n_{t}(x_{i})-\beta_{f}\cdot n_{f}(x_{i})]\;\;\;\;\Delta_{LP}(x_{i})=I(y_{i}=1)-P(y_{i}=1;\psi )+\lambda \cdot \left[\beta_{t}\cdot n_{t}(x_{i})-\beta_{f}\cdot n_{f}(x_{i})\right]$$

Intuitively, when a label for a given example has $\beta_{t}\cdot n_{t}\,\left(x_{i}\right)\gg \beta_{f}\cdot n_{f}\,\left(x_{i}\right)$ then the label is preferred by most of the advice for that example. Therefore, the gradient of that example will be increased. Conversely, when a label for a given example has $\beta_{b}\cdot n_{t}\,\left(x_{i}\right)\ll \beta_{f}\cdot n_{f}\,\left(x_{i}\right)$ then the label is avoided by most of the advice for that example and the gradient of that example will be decreased. Examples where there is no advice will have $\beta_{b}\cdot n_{t}\,\left(x_{i}\right)\approx \beta_{f}\cdot n_{f}\,\left(x_{i}\right)$ and the gradient will only be calculated from the data. In this way, we can also handle *conflicting advice for the same example*. We rewrite our gradients by setting η=α and λ=(1-α)/η to get

$$\eta \cdot \Delta_{LP}(x_{i})=\alpha \cdot (I(y_{i}=1)-P(y_{i}=1;\psi ))\;+(1-\alpha )\cdot \left[\beta_{t}\cdot n_{t}(x_{i})-\beta_{f}\cdot n_{f}(x_{i})\right]$$

**Figure 3:**
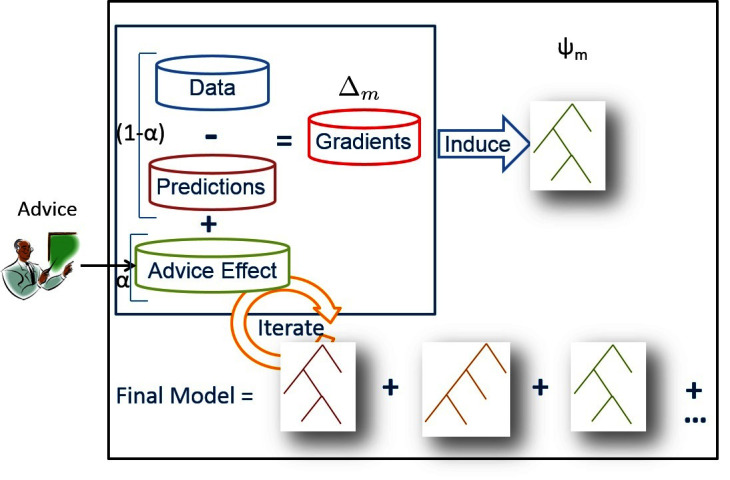
Standard RFGB is shown inside the black square (advice penalty equal to 0 for all examples) where relation regression trees are learned in a stage-wise fashion. When provided expert advice, the gradients for each example for which the advice applies are pushed in the direction of the advice (positive if the advice corresponds to the probability of the target being higher and vice versa). Figure appears in Odom et al. (2015b).

We learn RRTs stagewise as explained previously and shown in Figure 3. As shown in Algorithm 1, the strength of our approach not only comes from the concise way that our method is able to handle advice, but also that this advice is used to improve the model throughout the learning process. Using the advice just as the initial model will result in the learning algorithm undoing the advice if the examples are noisy. This can also be seen in our empirical evaluation.

**Algorithm 1 ARFGB: Advice for Relational Function Gradient Boosting (Odom et al., 2015b).**

**Table:** 

function AdviceBoost(D⁢a⁢t⁢a,A⁢d⁢v⁢i⁢c⁢e)
for1≤k≤Kdo ▷ Iterate through the predicates
for 1≤m≤Mdo ▷ Iteration through the gradient steps
$S_{k}$ =GenExamples (k,D⁢a⁢t⁢a,$F_{m-1}^{k}$,A⁢d⁢v⁢i⁢c⁢e)
$\Delta_{m}\,\left(k\right)$ =FitRelRegressTree ($S_{k}$,L)
$F_{m}^{k}=F_{m-1}^{k}+\Delta_{m}\,\left(k\right)$
end for
$P\left(Y_{k}=y_{k}\right|$Pa$\left(X_{k}\right))\propto \psi {}^{k}▷\psi^{k}$▹ $\psi^{k}$ is obtained by grounding $F_{M}^{k}$
end for
end function
function GenExamples (k,Data,F,Advice)
S=∅
for$1\leq i\leq N_{k}$do ▷ Iterate over all examples
Compute $P\left(y_{k}^{i}|x_{k}^{i},Pa\left(X_{k}^{i}\right)\right)▷$▹ Probability of the predicate being true
Compute $C^{i}=\beta_{t}\cdot n_{t}\,\left(x_{i}\right)-\beta_{f}\cdot n_{f}\,\left(x_{i}\right)$
$\begin{array}{r} r\Delta (y_{k}^{i},x_{k}^{i},Advice)=(1-\alpha )[I(y_{i}=1)-P(y_{k}^{i}=1)|Pa(x_{k}^{i}))]\\ +\alpha .C^{i} \end{array}$
$S=S⋃[\left(y_{k}^{i},x_{k}^{i},\Delta \left(y_{k}^{i},x_{k}^{i},Advice\right)\right]$
end for
return S
end function

More specifically, function GenExamples in Algorithm 1 computes both the probability of each example having a particular label and the impact of the advice on that particular label and example. Then the gradient of each example that is included in the training set is a combination of the gradient with respect to the data and the advice.

In the case of privileged information, we are learning two separate models. We compute the gradient for the standard features (i.e., $P\left(y_{i}|{𝐱}_{i}\right)$),

$$\frac{\partial MLL_{RP}(x)}{\partial \psi (x_{i};y_{i})}=\frac{\partial }{\partial \psi (x_{i};y_{i})}\log P(y_{i},x_{i};\psi )-\lambda \times KL(P_{D}(y_{i}|x_{i}^{RP})||P(y_{i}|x_{i}))\;\;\;\;\Delta_{RP}(x_{i})=I(y_{i}=1)-P(y_{i}=1;\psi )-\alpha \cdot \left(P(y_{i}=1|x_{i}^{CF})-P_{D}(y_{i}=1|x_{i}^{RP})\right)$$

Intuitively, if the learned distribution has a higher probability of an example belonging to the positive class compared to the 𝐑𝐏 distribution, $P(y_{i}=1|x_{i}^{CF})-P_{D}(y_{i}=1|x_{i}^{RP})$ would be positive and the gradient would be pushed lower. Hence the additional term would push the gradient (weighted by α) towards the true distribution as predicted by 𝐑𝐏. Thus, we are using the privileged features to guide the model to better generalization by biasing the algorithm to select features that make similar predictions to those made over the privileged features.

**Figure 4:**
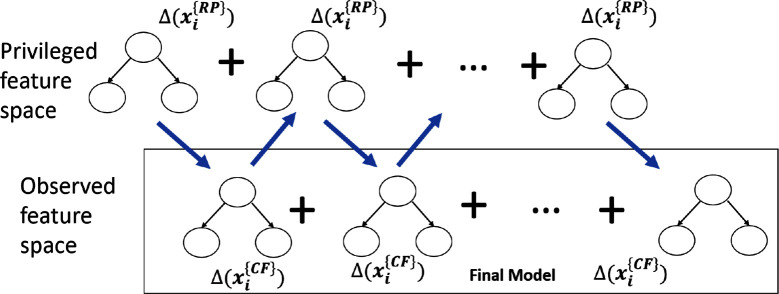
ARFGB-PI with privileged information. As can be observed, this is an iterative procedure. First, a small tree in the privileged feature space is learned. Next, a small tree in the classifier feature space is learned that combines the minimization of the error due to the data and the KL divergence to the privileged model. In the next step, a privileged tree is learned that minimizes the data gradients w.r.t. privileged features and the KL divergence to the observed features. This tree is added to the privileged model to calculate the gradients due to privileged features and the KL divergence. This process is repeated until convergence in the observed feature space.

The high-level idea is illustrated in Figure 4. The process is inherently iterative. A tree is first learned in the privileged space which is used to guide the learning in the observed feature space. Now the tree learned in the observed feature space will then be used to guide the next tree in the next privileged feature space along with the data. This process is repeated until convergence in the observed feature space. After learning the set of trees in the privileged feature space can be discarded while the trees from the observed space are treated as the final model.

The parameter α controls the influence of the privileged data on the learned distribution. In the extreme case of α=0, 𝐑𝐏 are completely ignored, i.e., 𝐑𝐏 is a noisy expert and we end up with the standard functional gradient. While it is potentially possible to choose α via some experimental method such as cross-validation, given the intuition that it represents the importance of privileged information, we define α for the classifier model^4^ as,

$$\alpha \propto \frac{\sum_{i}I(P_{D}(y_{i}=y_{i}^{*}|x^{RP})>=P(y_{i}=y_{i}^{*}|x^{CF}))}{N}$$

which is proportional to the fraction of the number of examples where the privileged features yield a better distribution over the observed labels ($y^{*}$) than the classifier features. As a better classifier model is learned, the value of α goes down giving more importance to the data.

###  3.4. Multiple Adaptations

Note that the objective function that we modified is a general one used in prior literature for relational domains. Thus we can use our knowledge-based RFGB for any model that uses RFGB. First, we discuss the generality of the approach by demonstrating how to use our advice for class-imbalance and qualitative constraints. Then, we show the versatility of our work in two directions: (1) what can be learned, and (2) what problems can be solved. To this effect, we show that we can learn the structure of both RDNs and MLNs, and that we can apply these models for relational policies and transfer learning.

####  3.4.1. What Relational Models Can Be Learned?

RDN: Based on prior work for RDNs (Natarajan et al., 2012), we can learn the structure of RDNs by learning a sequence of relational regression trees (RRT) for every predicate independently. This is due to the fact that the easiest way to learn a dependency network and consequently its relational extension is to simply learn a set of local conditional distributions and then combine them using Gibbs sampling. We boost each local distribution. We use the modified gradients to calculate the regression values for each ground example and use the modified regression examples to learn the RRTs.

MLN: There has been previous work on learning MLN as a series of RDNs (Khot et al., 2011). This is possible since MLNs make the conditional independence assumption only during learning, prior work (Khot et al., 2011) iteratively learned one tree for each predicate and used the previous trees for all the predicates for calculating the gradients. We also used the same approach with the modified gradients presented here.

####  3.4.2. What Relational Problems Can Be Solved?

Relational classification: By relational classification, we refer to classifying examples based on relational features. To this effect, RFGB learns a model (set of trees) to predict each class and then normalizes the resulting distribution to compute the posterior probabilities (Natarajan et al., 2012).

Relational policies: Imitation learning uses expert trajectories to directly learn a policy that best fits the expert trajectory. For relational domains, RFGB has been used to learn the relational policy by learning RRTs for every action (Natarajan et al., 2011). Since our trajectories can be noisy, we use advice to compute the modified gradients for every action to learn a knowledge-based relational policy.

Transfer learning: Transfer learning can be viewed as learning from a source task that has systematic differences with the target task. Previous work used RFGB to learn a model for a target domain by using the model from the source domain as an initial model (Natarajan et al., 2013) and refining the model with target examples. We, on the other hand, can learn a model from only source examples which can potentially be from a simpler domain. Expert advice can make source examples more applicable to target domain by focusing on key differences between source and target tasks.

##  4. Experiments

Given that our approach is a general framework for advice-giving to PLMs that encompasses different types of advice, we present empirical analysis that focuses on our two key directions:

(1) How effective are label preferences for noisy/uncertain data?

(2) Can we exploit privileged information to improve learning?

###  4.1. Results for Label Preferences

We present results for general label preferences, focusing on the breadth of problems (models and tasks) to which our framework can be applied. Then, we explore cost-based learning where there is a different cost for false positive and false negatives. Finally, we present initial results for qualitative constraints.

####  4.1.1. Preferential Advice

Our empirical evaluation of preferences aims to answer the following questions: (Q1) how effective is advice for relational classification, (Q2) can we employ advice in sequential problems (imitation learning), (Q3) does our method leverage knowledge across domains (transfer learning), and (Q4) how does advice help in standard domains for learning the popular PLM models (MLNs)? Each of these problem presents unique challenges where our advice-based framework can have significant impact.

For this set of experiments, we use two baseline approaches to compare against our method (presented as *ARFGB-LP*). To evaluate the importance of advice in our approach, we compare against RFGB without using any advice rules (*RFGB*). Also, we compare our approach against using the advice as initial model for RFGB (*Adv-Initial*) to demonstrate the effectiveness of our approach for incorporating advice. As the advice itself rarely specifies enough information to build a complete model, we show the relative influence of the advice in Table 1, which gives the fraction of training examples to which the advice applies. Although our advice only covers a part of the example space, the key intuition is that there are several small yet important regions where good advice might be crucial, which we also show empirically.

We show the advice across several tasks in Table 2. Note that the complete advice is stored as a decision-list of clauses. To compare the approaches, we use test-set accuracy averaged over multiple folds. We evaluate the results across three data sets. We use a different number of folds (4/5) in different experiments as they are natural for the particular domains.

**Table 1:** Appears in Odom et al. (2015b). The percentage of examples covered by each advice for tasks:

Domain	Domain	Drosophilia (RC)	Driving (IL)	Drosophilia (TL)	IMDB (RC using MLNs)
Advice Coverage	27.2	55.3	38.0	85.3

Relational Classification (RC) - Drosphilia; Imitation Learning (IL) - Driving; Transfer Learning (TL) - Drosophilia;

**Table 2:** Examples of label preference advice used in the respective experimental domains.

Domain	Advice Description	Preferred Label
Drosphilia (RC)	Cell regions with many neighbors and do not have a small perimeter or a particular color	Membrane
⟨numNeighbors(img,region,4),membrane,{mit,intra,extra}⟩		
Driving (IL)	If no car exists in front	Stay in lane
⟨¬in_front(carA,agent,time),stay,{moveRight,moveLeft}⟩		
Drosphilia (TL)	Cell regions with many neighbors	Membrane
⟨numNeighbors(img,region,4),membrane,{mit,intra,extra}⟩		
IMDB	Actors work under other people in the same movie	WorkedUunder
⟨movie(movie1,per1)∧movie(movie1,per2)∧genre(per2,g),workedUnder,¬workedUnder⟩		

##### Relational Classification:

We first evaluate our approach on relational classification in an image labeling domain where the goal is to label the segmented regions of an image. We use the Drosophila dataset (Cardona et al., 2010), which has 20 stacks of microscopic images of fruit flies ventral nerve cord. The possible labels include extra-cellular region, intra-cellular region, mitochondria, and membrane. This domain is naturally relational as the different regions have spatial relationships, e.g., membrane surrounds the cell (intra and mitochondria) and the number of segments can vary across images. We assume that we are given a perfectly segmented image (each region corresponds to one and only one object). Our features include region properties including color, color variance, area, perimeter, circularity, number of neighbors and edge features (representing boundary between regions) such as length, shape, area and color difference between regions.

We divide the dataset into 4 train and test sets, where each training set consists of 3 layers, while each test set consist of 2 layers that are 10 layers away from the training set. We introduced targeted noise in the membrane labels to emulate the natural mistakes in segmentation (for instance, missing small minute segments). Thus it is difficult to classify membrane regions in the noisy space. Our advice is then given on the noisy space to mimic a human expert.

In addition to relational models, we also compare against propositional models learned with a limited set of features (*Prop*) which do not have the spatial or relational neighborhood information, while the relational (*Rel*) models have the full feature set. We compare models learned without advice to ones with advice with our method (*ARFGB-LP*) and our baseline method (*Adv-Initial*). All the boosting approaches learn five trees in this domain.

In Table 3, we show the overall accuracy over all the labels where the label distribution is heavily skewed towards membrane. All advice methods are able to outperform those that did not have access to the advice. When comparing the advice models, our *ARFGB-LP* method is able to outperform the baseline *Adv-Initial* method. Even the *ARFGB-LP (Prop)* model which was learned from propositional feature space outperforms the *Adv-Initial (Rel)* method which had access to the neighborhood information. Recall that this is due to the *Adv-Initial* method starting with the advice and then refining the model away from the advice, while our *ARFGB-LP* method enables refining the models towards the advice at each step. Our method *ARFGB-LP* is able to combine information from both the training data and the advice model; thus, it achieves performance gains from both and answers Q1 affirmatively.

**Table 3:** Appears in Odom et al. (2015b). Relational Classification - Drosophila.

Model	ACC
FGB (Prop)	68.6 ± 0.4
RFGB (Rel)	69.3 ± 0.5
Adv-Initial (Prop)	68.8 ± 0.6
Adv-Initial (Rel)	91.6 ± 0.4
ARFGB-LP (Prop)	97.0 ± 0.5
ARFGB-LP (Rel)	**99.1** ± 0.4

##### 

##### Imitation Learning:

We next consider a sequential learning setting using the driving simulator extended from Judah et al. (2014). The goal in this domain is to navigate on a 5-lane highway, changing lanes in order to avoid other cars that are traveling at various speeds. The driver may either stay in the current lane, or move to the right or left lane. This domain is also relational as the spatial information is crucial to making driving decisions and the number of cars change across different scenarios. For example, if there is a car in front of an agent and a car to the right of the car in front, then moving into right lane might not be the correct action.

We learn from 10,000 training examples (100 trajectories) of an expert acting in the domain but choosing a sub-optimal action in certain states (like always driving in the left lane) and test on another 10,000 (100 trajectories) examples of another expert driving correctly. This is averaged over 5 runs. A sample advice could be to drive in the rightmost lane when possible, even though human drivers often prefer to drive in the left lanes. In our experiments, the advice was to stay in the current lane unless there are cars ahead.

The results, Table 4 show that our method is able to significantly outperform the work of Natarajan et al. (2011) which learns without advice. In sequential learning problems, we are able to successfully leverage data and advice to make more accurate predictions and thus positively answer Q2.

**Table 4:** Appears in Odom et al. (2015b). Imitation Learning - Driving.

Model	ACC
RFGB	52.5 ± 5.0
Adv-Initial	52.2 ± 5.9
ARFGB-LP	**96.0 **± 0.4

##### Transfer Learning:

We next verify how our advice framework can improve transfer learning in a relational domain. This could be especially useful if there is a related (possibly simpler) domain whose data points could be useful but the label space either has an incorrect distribution or the space is simply different (i.e., the domain contains similar features but different distributions or even different labels). Our advice can make the data in this form more applicable to the target domain, thus increasing the value of these examples for the target problem.

We use a version of the Drosophila dataset from the previous section. However, we assume that the source data has a limited label space. The source data is over whether a region is inside a cell or outside a cell. For our training data, we assume that intra-cellular and mitochondria regions are inside the cell, while extra-cellular and membrane regions are outside of the cell. However, the target problem is determining whether or not a region belongs to the mitochondria class. Notice that while the source data has a relationship with the target problem, it is not sufficient to build a model for mitochondria detection as there are a significant number of other objects inside a cell. Hence, advice is used to distinguish interesting (mitochondria) from non-interesting objects in the cell.

The results (shown in Table 5) show that the advice is able to filter out many of objects that are non-interesting (not mitochondria) and thus significantly improve the model. This is shown in the *ARFGB-LP* models improved accuracy on the non-interesting classes (other). While these results confirm that Q3 is true when the source and target domain share the same state space, it is an interesting future direction to show that a similar result can be achieved when transferring across different state spaces.

**Table 5:** Appears in Odom et al. (2015b). Transfer Learning - Drosophila.

Model	mit	other	total
FGB (Prop)	73.0	92.1	92.0± 0.9
RFGB (Rel)	79.7	91.7	91.6± 0.4
Adv-Initial (Prop)	84.3	90.1	90.0± 0.7
Adv-Initial (Rel)	86.3	90.3	90.3± 0.6
ARFGB-LP (Prop)	83.6	99.8	**99.7**± 0.2
ARFGB-LP (Rel)	75.2	99.5	**99.4**± 0.1

##### Learning MLNs

Finally, we also evaluate our advice framework in learning MLNs on a standard relational dataset, IMDB. The IMDB dataset (Mihalkova and Mooney, 2007) has information about movies, actors, and directors represented with five predicates: actor, director, genre, gender and workedUnder. We predict the workedUnder relationship between the people in this dataset. We performed five-fold cross-validation on this domain. We apply uniform noise (5, 10, 25%) to this dataset repeated five times for each fold and average the results. Unlike other domains, the IMDB dataset has a relatively large number of negative examples and hence even with 25% noisy positive examples, the impact on the negative examples is marginal. Hence, we also reduce the number of negative examples in all our approaches in this domain.

Table 6 shows the accuracy of our approaches on this domain for learning MLNs. Our approach (*ARFGB-LP*) outperforms learning without advice (*RFGB*), showing that our approach can be useful even when dealing with noisy examples and not just systematic noise. Also, it answers Q4 that advice can be used to learn MLNs.

**Table 6:** Appears in Odom et al. (2015b). Relational Classification - IMDB.

Model	5% Noise	10% Noise	25% Noise
RFGB	55.5 ± 9.3	55.9 ± 7.6	57.6 ± 9.0
ARFGB-LP	84.5 ± 6.0	81.0 ± 2.3	75.9 ± 4.4

#### 4.1.2. Cost-Based Learning

So far, we have considered the impact of advice on different types of tasks such as imitation learning, transfer learning and classification and on different types of models such as RDNs and MLNs. We now address the generality of the framework for different types of advice. To this effect, we first consider the common class-imbalance problem in relational problems. This occurs because there is a combinatorial explosion in the number of relationships between objects that are false. For instance, most people are not friends with each other, most authors do not publish together, most people do not own multiple houses. Consequently, there is an order-of-magnitude difference between the number of examples in one class vs another. While this problem has been addressed in propositional domains as *class-imbalance*, there is not much work inside the PLM community. More importantly, since most of the learning algorithms use conditional log-likelihood as the underlying optimization function, they tend to either ignore this problem or sub-sample one class to avoid the explosion. Recently, Yang et al. (2014) presented a more faithful approach of elicitation of the importance between the different classes and modified the objective function to include this advice while learning the boosted model. As we show in the previous section, our work can *theoretically* model this advice (class-imbalance) as preferential advice. In this section, we evaluate it empirically by answering the following questions: (Q1) can our method faithfully incorporate advice on class-imbalance and (Q2) how does our method compare to state-of-the-art learning methods that correct class-imbalance? We compare our method (*ARFGB-CI*) to several baselines including learning an *MLN* (Khot et al., 2011), learning a single relational regression tree (*RRT*) and learning 20 trees using *RFGB*. We also compare our method to a previous method Soft-Margin Relational Functional Gradient Boosting (*SMRFGB*) by Yang et al. (2014) which alters standard RFGB to account for skewed data. Note that while they showed a range of different parameter settings, we compare to the best (or near-best) from among all the parameters settings. By comparing to this specialized method, we demonstrate the versatility of our approach. Accuracy is not used as a performance metric in this section as class-imbalance makes this metric less informative. This is due to the fact that methods that predict all examples as the majority class can achieve very high performance without learning anything useful. Instead, we focus on problems where correctly predicting the positive minority class outweighs the negative class. To this effect, we follow previous work (Weng and Poon, 2008; Yang et al., 2014) and use weighted-auc that weights the high-recall areas of the ROC curve more than the low-recall areas. This makes sense in domains like medical predictions where it is extremely costly to not predict a heart attack but is less costly to predict a heart attach where one is not likely to occur. Similarly, we compare weighted f-score and the false negative rate. We refer to the previous work on class-imbalance (Yang et al., 2014) for more details on these evaluation measures. We perform experiments in several standard relational domains including cora (Poon and Domingos, 2007) - a publication and citation dataset, heart - a heart attack dataset, uw (Mihalkova and Mooney, 2007) - a dataset of professors and students, and webkb (Richardson et al., 2006) - a course dataset. All of these experiments have parameters set as 0.9 for α, 4.0 for $\beta_{t}$ and 0.0 for $\beta_{f}$. The optimal parameters settings may depend on the skew of the dataset among other factors, but these were selected as well performing parameters across all domains. The results, shown in Table 7, clearly demonstrate that our method is able to outperform standard learning methods that do not incorporate advice. Our method captures advice about class-imbalance as well as the state-of-the-art method for PLMs by Yang et al. (2014). This result answers Q1 and Q2 both positively as we are comparable or better with the best alternative for PLMs.

**Table 7:** Appears in Odom et al. (2015b). Results for Class-Imbalance advice.

	MLN	RRT	RFGB	SMRFGB	ARFGB-CI
cora					
WAUC	0.233	0.709	0.723	0.712	0.717
FNR	0.626	0.663	0.481	0.0	0.02
WF	0.832	0.867	0.864	0.974	0.957
heart					
WAUC	0.026	0.302	0.295	0.353	0.324
FNR	0.386	0.641	0.354	0.0	0.09
WF	0.569	0.351	0.622	0.91	0.843
uw					
WAUC	0.044	0.765	0.886	0.892	0.924
FNR	0.047	0.113	0.006	0.0	0.017
WF	0.701	0.877	0.95	0.733	0.935
webkb					
WAUC	0.004	0.484	0.489	0.466	0.469
FNR	0.43	0.612	0.501	0.0	0.0
WF	0.429	0.378	0.451	0.742	0.79

#### 4.1.3. Qualitative Constraints

We now consider the final type of advice that is most commonly used in probabilistic learning systems called qualitative advice (Altendorf et al., 2005; Yang and Natarajan, 2013). More concretely, we consider providing monotonicity advice between the features and the target predicates. For example, in medicine, it is useful to provide an advice such as “increase in blood pressure increases the risk of heart attack”. Note that this type of advice is not providing any quantitative assertion about the probabilistic interactions but merely points out the qualitative relationship between blood pressure and risk of heart attack. To be precise, this statement specifies how the probability mass function shifts in the conditional table. Previous work have demonstrated the effectiveness and efficiency of this type of advice when learning propositional probabilistic models such as Bayes nets (Altendorf et al., 2005; Yang and Natarajan, 2013). There is not any work on using such knowledge for richer models such as PLMs for us to compare as baselines. 

Consequently, to further empirically evaluate the generality of our approach, we consider qualitative advice in this section. Specifically, we focus on the following questions: (Q1) can our framework incorporate more expressive advice, and (Q2) what is the significance of this type of advice? As mentioned earlier, there are few methods that can incorporate monotonic advice in PLMs. So we compare it against our method that incorporates simple label preferences. One way to understand monotonicity is that the more general the advice, the coarser it is likely to be. For instance, simply stating high BP causes higher risk of heart attack is more general than specifying that as BP increase, the higher the risk of heart attack. The first case simply states a label preference for a particular region of the feature space but the second is more informative in explaining how the label changes over the region of features. The proposed approach for monotonicities in this paper is of the latter case (*ARFGB-Mono* in Table 8) while the alternative general advice (*ARFGB-LP* in Table 8) is the former case. As a standard baseline, we employ *RFGB* without any advice.

**Table 8:** Appears in Odom et al. (2015b). Results for monotonicity advice

	RFGB	ARFGB-PI	ARFGB-Mono
ACC	64.0	74.9	82.0

As a proof of concept, our test domain is a synthetic heart attack prediction domain. It is created with high cholesterol as an indicator of a heart attack. The higher the cholesterol, the higher the chance of having a heart attack. The models were learned from small training sets (50 examples) and tested on much larger training sets (5,000 examples). We repeated the experiment 10 times and report the average accuracy. The results, shown in Table 8, show that our method incorporating the monotonic advice significantly outperforms all other methods. As expected, *AFRGB-LP* outperforms not incorporating any advice. However, it can not take full advantage of the relationship between blood pressure and heart attacks. As shown, Q1 and Q2 are answered affirmatively.

### 4.2. Results for Privileged Information

We now discuss the results when learning with privileged features. Our evaluation focuses on a NLP dataset of football games.

The NFL dataset (Natarajan et al., 2014) contains information about games during the 1995–2000 seasons. We consider two problems from this dataset: (1) learn from home games (seasons 1995–1997) of a particular team to predict winners all games of that team (seasons 1998–2000) and (2) learn from homes games of all team in a particular division (1995–1997) to predict the winner of all games played by that division (1998–2000). These two problems allow for investigation of the impact of privileged information with different quantities of training data.

We define natural privileged information in this domain. The 𝐂𝐅 features include information about when the game was played, if the game is regular or post season, the number of turnovers/yards and whether the final score was within one possession. The 𝐑𝐏 features included the number of points (discretized to multiples of seven so it is not a perfect predictor) and which team had more turnovers. The aim is to use the privileged information to generalize from home games of three seasons to all games of subsequent seasons. As these features represent high-quality privileged information, the α value when learning the privileged model is 0. The proportionality constant for α when learning the classifier model is $\frac{1}{2}$.

**Figure 5:**
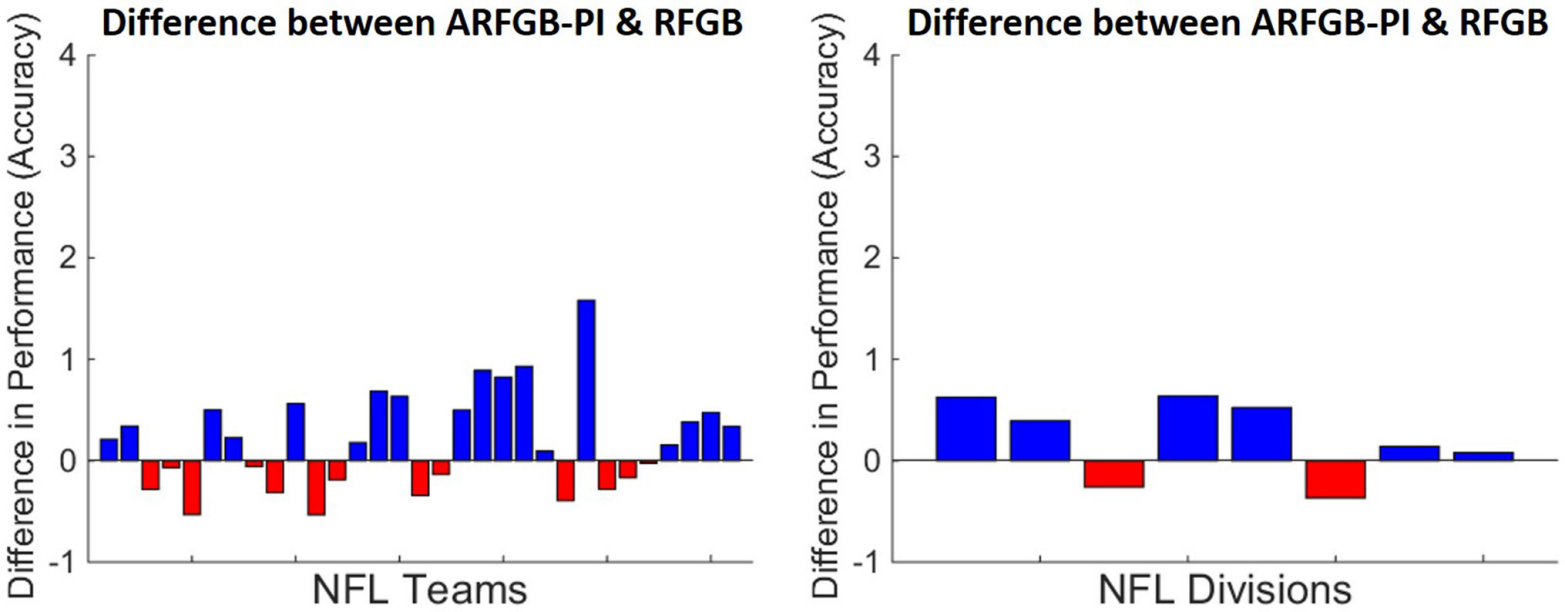
Following Sharmanska et al. (2013), we show a direct comparison between the *ARFGB-PI* and its standard learner counterparts (*RFGB*). Each bar represents the difference between the privileged and standard learner. The NFL dataset for individual teams (LEFT) and divisions (RIGHT) are shown separately. Note that the blue bars indicate the datasets where the privileged learner outperforms the standard learner and red bars indicate the inverse.

Figure 5 shows the difference between *ARFGB-PI* (with privileged information) and *RFGB* training and testing on individual NFL teams. This dataset is particularly challenging as teams could have varying success in different seasons. The goal is to generalize from a teams home games to all their games in subsequent seasons. Note that predictions for 18 of 31 teams are improved with the use of privileged information.

The results are shown on all home games from teams in a division (4 teams to a division) and testing on all of those teams subsequent games. Even with an increase in number of training examples, *ARFGB-PI* (the relational functional-gradient boosting with privileged information) is able to outperform *RFGB* (the standard functional-gradient boosting) for 6 of the 8 NFL divisions. Experiments on the NFL domain suggest that our privileged approach *ARFGB-PI* can improve performance in noisy, real-world tasks.

###  4.3. Discussion

The experimental results demonstrate a few aspects of probabilistic logic models - first is that they are capable of employing rich human inputs beyond simple labelled examples. Second is that, the gradient boosting technique for learning PLMs makes it possible for developing a unified framework based on preferences. Third, the results clearly show that the preference based learning method is indeed useful across many real tasks. It must be mentioned that while the results of using privileged information is not as impressive as the other forms of advice, this is consistent with the results from the original privileged information framework (Vapnik and Izmailov, 2015). Note that the key difference between the privileged information and the preference based framework is that in the latter, the advice is employed as a constraint which if violated incurs a penalty. Privileged information on the other hand is merely used to guide the examples. It is interesting direction to combine the two frameworks which we intent to pursue in the future and is beyond the scope of the current work.

##  5. Conclusion and Future Work

We propose a novel method for allowing rich interaction between experts and PLMs. The key insight of our approach is that it continuously leverages advice provided by domain experts while learning as against traditional methods that consider it as an initial model. The approach obtains advice in the natural form of first-order logic that goes beyond specific examples and allows for advice that is generalizable across several examples/situations. We showed that the generality of our formalism by adapting it for four types of advice - preference-based, cost-sensitive, qualitative advice and privileged information. Empirically, we show that our approach is effective when learning with the proposed advice from noisy training examples or when dealing with few examples. Our framework allows for controlling the magnitude of an individual piece of advice as well as the relative impacts of advice and training data on the learned model.

There are several possible extensions for future work. First, we will work on providing a theoretical understanding of our update to the gradients. Second, currently our approach considers all advice to be equal, but this may not always be the case. We will work on associating weights with the expert advice which can either be learned or provided by the expert. Next, while we have some initial results suggesting that the trade-off between advice and data is robust to reasonable values of α, we propose to study the impact of this trade-off with noisy advice. Extending this work to other types of relational problems such as planning, sequential decision-making and possibly inverse reinforcement learning is a fruitful direction. Finally, we assume all the advice to be given before learning commences. Relaxing this assumption to provide advice as necessary (in an active manner) if an interesting area of future research.

## Author Contributions

Both authors contributed to the writing of this article. In addition, PO obtained the empirical results.

## Conflict of Interest Statement

The authors declare that the research was conducted in the absence of any commercial or financial relationships that could be construed as a potential conflict of interest.

## Footnotes

1 This work extends our previous work (Odom et al., 2015b) by generalizing the advice, and providing more types of advice for class-imbalance and qualitative constraints. We also provide new experiments for these advice. The work on label preferences and privileged information also appears in Odom (2017).

2 It is trivial to extend this definition to define a β_*t*_ and β_*f*_ for each advice rule separately by defining this to be a vector.

3 For instance in many medical diagnosis tasks, it is important sometimes to reduce false negatives even at the cost of introducing a few false positives.

4 α can be likewise defined for the privileged model.
